# Effect of weekend catch-up sleep on high-sensitivity C-reactive protein levels according to bedtime inconsistency: a population-based cross-sectional study

**DOI:** 10.1038/s41598-022-25787-x

**Published:** 2022-12-14

**Authors:** Soyoung Park, Dong Yoon Kang, Hyungwoo Ahn, Namwoo Kim, Jeong-Hwa Yoon, Bo Ram Yang

**Affiliations:** 1 Department of Family Medicine, Millennium Seoul Internal Medicine Clinic and Health Care Center, Inchon, Republic of Korea; 2 Department of Preventive Medicine, Ulsan University Hospital, Ulsan, Republic of Korea; 3 Department of Radiology, Seoul National University Bundang Hospital, Seongnam, Republic of Korea; 4 Department of Neuropsychiatry, Seoul National University Hospital, Seoul, Republic of Korea; 5 Institute of Health Policy and Management, Medical Research Center, Seoul National University, Seoul, Republic of Korea; 6 College of Pharmacy, Chungnam National University, Daejeon, 34134 Republic of Korea

**Keywords:** Epidemiology, Inflammatory diseases, Risk factors

## Abstract

To investigate the associations of weekend catch-up sleep (WCS) and high-sensitivity C-reactive protein (hs-CRP) levels according to bedtime inconsistency in the Korean population. In this cross-sectional study using the Korea National Health and Nutrition Examination Survey (2016–2018) with 17,665 participants, four groups were defined: no-WCS (WCS within ± 1 h of weekday sleep time), moderate WCS (1 ≤ , < 3 h), severe WCS (≥ 3 h), and inverse WCS (≤ − 1 h). An inconsistent bedtime was defined as a > 2 h difference between weekend and weekday bedtimes. Outcomes were divided into quartiles based on the hs-CRP level: Lowest (< 0.34), Middle-low (≥ 0.34, < 0.55), Middle-high (≥ 0.55, < 1.10), Highest (≥ 1.10). Adjusted odds ratios (aORs) with 95% confidence intervals (CIs) were calculated using multinomial logistic regression, controlling for relevant covariates. Moderate WCS was associated with a lower risk for the highest hs-CRP levels than no WCS (aOR = 0.87, 95% CI 0.78–0.97), and a similar association was observed only in participants with consistent bedtimes (aOR = 0.88, 95% CI 0.78–0.99). Significant interactions of those associations of WCS and hs-CRP levels with bedtime inconsistency were found. These findings provide evidence that people with inconsistent bedtimes would have limited protective effect of WCS on hs-CRP.

## Introduction

Sleep is an essential element for maintaining physical and mental function. Many studies have indicated that aspects of sleep, such as sleep duration, timing of sleep, quality of sleep, and sleep efficiency, are associated with inflammation, metabolism, and the cardiovascular system^1^. Extensive research has explored the link between sleep duration and inflammation. Experimental studies have shown associations between sleep deprivation and inflammation parameters (interleukin-6 [IL-6], C-reactive protein [CRP], and tumor necrosis factor-α)^2–4^, and epidemiological studies have reported that sleep restriction or excessive sleep duration were associated with elevated levels of inflammation^3,5,6^.

CRP, which is widely used as an inflammation marker, is synthesized in the liver in response to inflammatory cytokines such as IL-6 and plays a well-known role in predicting risk for cardiovascular disease^7,8^. In particular, high-sensitivity-CRP (hs-CRP) is advantageous for use in the general population, as it can provide a more accurate assessment of microvascular levels of CRP. A previous study reported that hs-CRP levels were associated with sleep disorders such as insomnia^9^. Moreover, several studies have shown associations of sleep characteristics including deprivation or excessive sleep, with increased levels of hs-CRP in western^10–12^ and Asian^13^ general populations.

In contemporary society, sleep patterns during the weekdays and weekends can be different; for example, people may engage in catch-up sleep or sleep less on the weekend. Compared to sleep duration, relatively few studies have been conducted to evaluate the health effects of weekend catch-up sleep (WCS), which compensates for the lack of sleep during the weekdays. However, some recent studies have suggested that adequate recovery sleep (e.g., WCS) could be beneficial for dyslipidemia, hypertension, quality of life, obesity, and inflammation^5,14–18^ due to the possibility that catch-up sleep might normalize elevated levels of inflammation markers due to sleep deprivation^19^. Despite the wide variation in sleep patterns on weekdays and weekends, limited studies have interpreted the health effects of WCS in the context of other sleep patterns, including sleeping less on the weekend and bedtime inconsistency.

In this study, we investigated the associations of various weekday and weekend sleep patterns, including WCS and sleeping less on the weekend, with hs-CRP levels using data from a large population-based cross-sectional study in Korea. Furthermore, we evaluated the combined effects of WCS and bedtime consistency on hs-CRP levels.

## Methods

### Data source

We used data from the seventh Korea National Health and Nutrition Examination Survey (KNHANES VII), which was conducted in South Korea from 2016 to 2018. The KNHANES, an ongoing, nationally representative, cross-sectional, stratified, complex, and multistage sample survey, was initiated in 1998 by the Korea Centers for Disease Control and Prevention (KCDC) as an annual survey to assess the population’s health and nutritional status. The KNHANES data now include information on socioeconomic status, health-related behaviors, quality of life, healthcare utilization, anthropometric measures, biochemical and clinical profiles for non-communicable diseases, and dietary intake of approximately 10,000 individuals each year. A detailed description of the KNHANES has been published elsewhere^20^.

The present study was exempted from review by the Institutional Review Board of Seoul National University Hospital (IRB number: E-2008-049-1147). All the KNHANES VII are conducted with the participants’ consent. All methods were carried out in accordance with KNHANES guidelines and regulations^21^.

### Study subjects

In this population-based, cross-sectional study, we included participants aged 12 and older without any missing values or unknown/no response for sleep time on weekends or weekdays and hs-CRP levels from the KNHANES VII. Participants who reported extremely long (1000 min or longer per day) or short (60 min or shorter per day) sleep duration were excluded. The proportions of participants with missing data for sleep time on weekends, weekdays, and hs-CRP levels in the KNHANES VII data were 9.65%, 9.65%, and 22.27%, respectively.

### Sleep duration, weekend catch-up sleep, and bedtime consistency

Sleep duration was calculated based on self-reported bedtime and wake-up times on weekdays and weekends. This information was elicited using the following questions: “On average, what time do you go to sleep and wake up on a weekday (workday)?” and “On average, what time do you go to sleep and wake up on the weekend (a day off or the day before a day off)?”, to which participants gave responses in terms of hours and minutes. WCS duration was calculated as the average weekend sleep duration per day minus the average weekday sleep duration per day. We classified the study population into the following four groups using WCS duration: no-WCS (WCS ± 1 h relative to the weekday sleep duration), moderate WCS (1 ≤, < 3 h of WCS), severe WCS (≥ 3 h of WCS), and inverse WCS, which corresponded to those who slept less on the weekend (≤ − 1 h of WCS).

In a stratified analysis, participants were classified by the consistency of their bedtimes between weekends and weekdays apart from sleep duration. An inconsistent bedtime was defined as a difference between weekend and weekday bedtimes of more than 2 h, and others were considered as consistent bedtime.

### Measurement of hs-CRP levels

Blood samples were collected from participants aged 10 years and over after at least 8 h of fasting in the morning (about 6:00 am to noon), and processed, immediately refrigerated, and transported in cold storage at 2–8 °C to a central laboratory (Seegene Medical Foundation, Seoul, South Korea). The serum hs-CRP level was measured by immunoturbidimetry using a Cobas device with a Roche cardiac hs-CRP test (Roche, Mannheim, Germany). The minimum value in the specimens was 0.15 mg/L, and the maximum value was 20 mg/L. To facilitate a proper interpretation of our results in comparison with prior studies, and to explore in detail the associations with the risk of elevated CRP levels, the level of hs-CRP was divided into quartiles: lowest (first quartile), middle-low (second quartile), middle-high (third quartile) and high (fourth quartile)^15,22–24^.

### Covariates

We obtained information on sociodemographic (age, sex, household income, marital status, and employment status) and health-related variables (body mass index [BMI], perceived stress, the frequency of binge alcohol consumption, smoking status, medium-intensity physical activity, high risk for cardiovascular disease [CVD], and cancer) from health examinations and the self-reported survey. Household income was categorized into quartiles, from quartile 1 (low) to quartile 4 (high), and employment status was categorized as employed, unemployed or economically inactive (e.g., students, retirees, and housewives), not applicable (< 15 years of age), or unknown/no response. Height was measured using a seca 225 device (seca, Germany), and body weight was measured using a GL-6000-20 device (G-tech, Korea). Self-reported stress (responses to the question, “How much stress do you feel in your daily life?”) was classified as extreme, very, somewhat, a little, and unknown/no response. The frequency of binge alcohol consumption (≥ 7 drinks in one session) was categorized as occasional (< 1 time/week), frequent (≥ 1 time/week), or unknown/no response. Participants were classified by smoking status as non-smokers, ex-smokers, current smokers, or unknown/no response. To identify high-risk patients at high risk for CVD, information was obtained regarding hypertension, diabetes mellitus, dyslipidemia, stroke, myocardial infarction, and angina diagnosed by a doctor. Participants were considered high-risk if they had at least one diagnosis of these diseases.

### Statistical analysis

Baseline characteristics were presented as mean ± standard deviation (SD) for continuous variables and as frequency and proportion for categorical variables. For comparisons between WCS groups, analysis of variance was used for continuous variables, and the chi-square test was used for categorical variables. Multinomial logistic regression was performed to examine associations between the WCS groups and the four hs-CRP groups, of which the lowest (first quartile) served as the reference group. The odds ratios (ORs) and 95% confidence intervals (CIs) were adjusted for covariates. The covariates included in the final models included age, sex, household income, marital status, economic activity, BMI, perceived stress, the frequency of binge alcohol consumption, smoking status, medium-intensity physical activity, high-risk status for CVD, and cancer. We assessed the interaction between WCS and bedtime inconsistency by using an interaction term in a multinomial model, followed by stratified analyses according to bedtime inconsistency. The likelihood ratio test was used to test hypotheses of significance. Considering statistical and clinical significance, subgroup analyses based on sleep duration (≤ 6 h, 6–8 h, ≥ 8 h) and BMI (normal weight [< 25 kg/m^2^] vs. obesity [≥ 25.0 kg/m^2^]) were conducted. Since sleep needs and inflammation are related to age, and elevated CRP levels are also associated with aging-related diseases, a subgroup analysis according to age group (< 40 years, 40–64 years, and ≥ 65 years) was conducted. As a sensitivity analysis, we performed a regression analysis of the associations between WCS and the logarithm of the hs-CRP level.

All statistical analyses were performed with SAS software (version 9.4; SAS, Institute, Inc., Cary, NC, USA).

## Results

**Figure 1:**
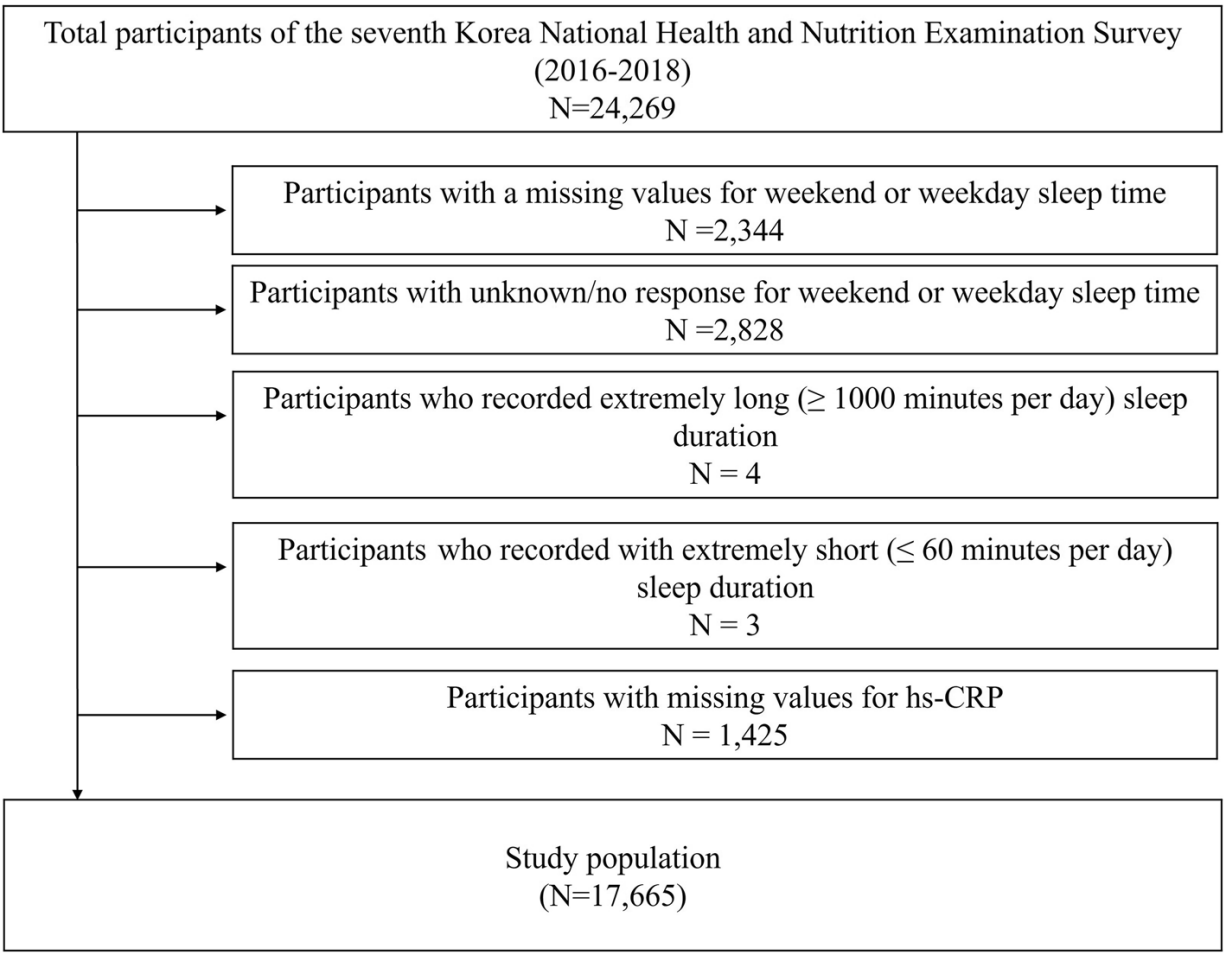
Flowchart of the study population. *CRP* C-reactive protein.

Of the 24,269 participants in the KNHANES VII, we excluded the following participants: (1) participants with missing values for sleep time on weekends or weekdays (n = 2344), (2) participants with unknown/no response for weekend or weekday sleep time (n = 2828), (3) participants with an extremely long duration of sleep (1000 min or longer per day) or an extremely short duration (60 min or shorter) (n = 7), and (4) participants with missing values for hs-CRP levels (n = 1425). Finally, 17,665 participants were included in the analysis (Fig. 1).

The baseline characteristics of participants in the four WCS groups are presented in Table 1. Age was significantly differed among the four groups, and was markedly lowered in the severe WCS group (mean ± SD: 34.39 ± 16.63 years). The no-WCS group had the highest proportion of participants in the lowest quartile of household income level, employment, prevalence of chronic diseases, and cancer. The severe WCS group tended to have a higher physical activity level and were less likely to be married. Total, weekday, and weekend sleep duration were different among the 4 groups (Table 1).

**Table 1:** Baseline characteristics of the four groups according to weekend catch-up sleep. All values are expressed as n (%) unless otherwise indicated.

Characteristics	No-WCS (n = 10,309)	Moderate WCS (n = 5044)	Severe WCS (n = 1355)	Inverse WCS (n = 957)	*p-*value
Age, mean ± SD	54.25 ± 17.68	41.12 ± 16.29	34.39 ± 16.63	46.79 ± 18.01	< 0.001
**Age group**					
< 20	402 (3.90)	646 (12.81)	359 (26.49)	68 (7.11)	< 0.001
≥ 20 and < 40	1942 (18.84)	1646 (32.63)	471 (34.76)	300 (31.35)	
≥ 40 and < 65	4488 (43.53)	2356 (46.71)	464 (34.24)	410 (42.84)	
≥ 65 and over	3477 (33.73)	396 (7.85)	61 (4.50)	179 (18.70)	
**Sex**					
Men	4698 (45.57)	2197 (43.56)	621 (45.83)	432 (45.14)	0.112
Women	5611 (54.43)	2847 (56.44)	734 (54.17)	525 (54.86)	
BMI, mean ± SD	24.04 ± 3.52	23.54 ± 3.69	23.21 ± 3.92	24.18 ± 3.79	< 0.001
**Household income level**					
Low	2432 (23.66)	486 (9.65)	129 (9.56)	155 (16.2)	< 0.001
Medium–low	2563 (24.94)	1131 (22.46)	332 (24.59)	240 (25.08)	
Medium–high	2607 (25.37)	1562 (31.02)	437 (32.37)	315 (32.92)	
High	2675 (26.03)	1857 (36.87)	452 (33.48)	247 (25.81)	
**Marital status**					
Yes	8783 (85.2)	3474 (68.87)	652 (48.12)	731 (76.38)	< 0.001
No	1526 (14.8)	1570 (31.13)	703 (51.88)	226 (23.62)	
**Employment status**					
Employed	5448 (52.85)	3258 (64.59)	833 (61.48)	588 (61.44)	< 0.001
Unemployed or economically inactive	4657 (45.17)	1535 (30.43)	429 (31.66)	333 (34.80)	
Not applicable (< 15 year)	196 (1.9)	250 (4.96)	93 (6.86)	35 (3.66)	
Unknown/no response	8 (0.08)	1 (0.02)	0 (0)	1 (0.1)	
**Degree of perceived stress**					
Extreme	460 (4.46)	203 (4.02)	78 (5.76)	61 (6.37)	< 0.001
Very	2106 (20.43)	1185 (23.49)	384 (28.34)	227 (23.72)	
Somewhat	5665 (54.95)	2989 (59.26)	734 (54.17)	511 (53.40)	
A little	2042 (19.81)	662 (13.12)	156 (11.51)	156 (16.30)	
Unknown/no response	36 (0.35)	5 (0.1)	3 (0.22)	2 (0.21)	
**Smoking status**					
Non-smoker	6210 (60.24)	3397 (67.35)	904 (66.72)	583 (60.92)	< 0.001
Ex-smoker	2362 (22.91)	845 (16.75)	165 (12.18)	154 (16.09)	
Current smoker	1707 (16.56)	796 (15.78)	283 (20.89)	219 (22.88)	
Unknown/no response	30 (0.29)	6 (0.12)	3 (0.22)	1 (0.1)	
**Binge alcohol consumption**					
Occasional (< 1 time/week)	5196 (50.4)	2855 (56.6)	719 (53.06)	487 (50.89)	< 0.001
Frequent (≥ 1 time/week)	1701 (16.5)	843 (16.71)	235 (17.34)	188 (19.64)	
Unknown/no response	3412 (33.1)	1346 (26.69)	401 (29.59)	282 (29.47)	
**Medium-intensity physical activity**					
Yes	633 (6.14)	485 (9.62)	164 (12.1)	86 (8.99)	< 0.001
No	9467 (91.83)	4201 (83.29)	986 (72.77)	841 (87.88)	
Unknown/no response	209 (2.03)	358 (7.1)	205 (15.13)	30 (3.13)	
**Sleep duration, mean ± SD**					
Total	7.23 ± 1.36	7.25 ± 1.12	7.05 ± 1.3	7.41 ± 1.25	< 0.001
Weekday	7.22 ± 1.36	6.81 ± 1.13	5.95 ± 1.35	7.82 ± 1.28	< 0.001
Weekend	7.26 ± 1.37	8.35 ± 1.17	9.81 ± 1.48	6.39 ± 1.31	< 0.001
**Bedtime inconsistency**					
Consistent	9853(95.58)	4498(89.18)	1045(77.12)	555(57.99)	< 0.001
Inconsistent	456(4.42)	546(10.82)	310(22.88)	402(42.01)	
**High risk for CVD**					
Yes	4280 (41.52)	1019 (20.2)	195 (14.39)	312 (32.6)	< 0.001
No	5679 (55.09)	3436 (68.12)	833 (61.48)	595 (62.17)	
Unknown/no response	350 (3.4)	589 (11.68)	327 (24.13)	50 (5.22)	
**Cancer**					
Yes	604 (5.86)	152 (3.01)	23 (1.7)	39 (4.08)	< 0.001
No	9355 (90.75)	4303 (85.31)	1005 (74.17)	867 (90.6)	
Unknown/no response	350 (3.4)	589 (11.68)	327 (24.13)	51 (5.33)	

*WCS* weekend catch-up sleep, *BMI* body mass index, *SD* standard deviation, *CVD* cardiovascular disease.

Compared to the no-WCS group, the moderate WCS group had a lower risk of being in the higher quartiles of hs-CRP levels in the multinomial regression model (aOR = 0.89, 95% CI 0.80–0.99 for middle-high hs-CRP levels, aOR = 0.87, 95% CI 0.78–0.97 for the highest hs-CRP levels). Severe WCS was not significantly associated with higher quartiles of hs-CRP levels (aOR = 0.99, 95% CI 0.83–1.18 for middle-high hs-CRP levels; aOR = 1.04, 95% CI 0.87–1.25 for the highest hs-CRP levels). A higher risk for middle-low hs-CPR levels was shown in the inverse WCS group (aOR = 1.22; 95% CI 1.00–1.48), but this was not the case for middle-high hs-CRP levels (aOR = 1.02, 95% CI 0.83–1.25) or the highest hs-CRP levels (aOR = 1.03, 95% CI 0.84–1.28). We conducted a stratified analysis by sex. The point estimate of the risk for the higher quartiles tended toward the null in men. The risks in women were similar to those found in the main analysis (Table 2).

**Table 2:** Associations between weekend catch-up sleep and high-sensitivity C-reactive protein levels.

	Crude OR (95% CI)	*P* value	Adjusted OR^a^ (95% CI)	*p-*value
**Total (n = 17,665)**				
Middle-low hs-CRP level (vs. lowest)				
No-WCS group	Reference		Reference	
Moderate WCS group	0.79 (0.72,0.87)	< 0.001	0.96 (0.87,1.07)	0.457
Severe WCS group	0.73 (0.62,0.85)	< 0.001	1.01 (0.85,1.19)	0.945
Inverse WCS group	1.15 (0.95,1.38)	0.164	1.22 (1.00,1.48)	0.049
Middle-high hs-CRP level (vs. lowest)				
No-WCS group	Reference		Reference	
Moderate WCS group	0.65 (0.59,0.72)	< 0.001	0.89 (0.80,0.99)	0.028
Severe WCS group	0.59 (0.51,0.70)	< 0.001	0.99 (0.83,1.18)	0.916
Inverse WCS group	0.93 (0.77,1.13)	0.473	1.02 (0.83,1.25)	0.874
Highest hs-CRP level (vs. lowest)				
No-WCS group	Reference		Reference	
Moderate WCS group	0.59 (0.53,0.65)	< 0.001	0.87 (0.78,0.97)	0.010
Severe WCS group	0.59 (0.50,0.69)	< 0.001	1.04 (0.87,1.25)	0.657
Inverse WCS group	0.95 (0.78,1.15)	0.580	1.03 (0.84,1.28)	0.753
**Men (n = 7948)**				
Middle-low hs-CRP level (vs. lowest)				
No-WCS group	Reference		Reference	
Moderate WCS group	0.92 (0.79,1.06)	0.245	1.08 (0.92,1.27)	0.366
Severe WCS group	0.88 (0.69,1.11)	0.286	1.13 (0.88,1.47)	0.337
Inverse WCS group	1.15 (0.86,1.55)	0.347	1.19 (0.88,1.62)	0.263
Middle-high hs-CRP level (vs. lowest)				
No-WCS group	Reference		Reference	
Moderate WCS group	0.70 (0.60,0.81)	< 0.001	0.92 (0.78,1.08)	0.289
Severe WCS group	0.60 (0.47,0.77)	< 0.001	0.92 (0.70,1.21)	0.546
Inverse WCS group	0.89 (0.66,1.20)	0.456	0.95 (0.70,1.31)	0.767
Highest hs-CRP level (vs. lowest)				
No-WCS group	Reference		Reference	
Moderate WCS group	0.66 (0.57,0.76)	< 0.001	0.96 (0.81,1.13)	0.608
Severe WCS group	0.66 (0.52,0.84)	0.001	1.13 (0.87,1.48)	0.364
Inverse WCS group	0.93 (0.69,1.24)	0.609	1.03 (0.75,1.41)	0.868
**Women (n = 9717)**				
Middle-low hs-CRP level (vs. lowest)	Reference		Reference	
No-WCS group	0.72 (0.64,0.81)	< 0.001	0.89 (0.78,1.02)	0.089
Moderate WCS group	0.63 (0.51,0.77)	< 0.000	0.93 (0.74,1.16)	0.511
Severe WCS group	1.14 (0.89,1.46)	0.309	1.22 (0.95,1.58)	0.119
Inverse WCS group				
Middle-high hs-CRP level (vs. lowest)				
No-WCS group	Reference		Reference	
Moderate WCS group	0.63 (0.56,0.72)	< 0.001	0.88 (0.77,1.02)	0.082
Severe WCS group	0.60 (0.48,0.74)	< 0.001	1.07 (0.84,1.36)	0.577
Inverse WCS group	0.97 (0.75,1.25)	0.792	1.08 (0.82,1.42)	0.573
Highest hs-CRP level (vs. lowest)				
No-WCS group	Reference		Reference	
Moderate WCS group	0.55 (0.48,0.62)	< 0.001	0.81 (0.70,0.94)	0.006
Severe WCS group	0.53 (0.42,0.65)	< 0.001	0.97 (0.75,1.25)	0.821
Inverse WCS group	0.96 (0.74,1.25)	0.780	1.06 (0.80,1.40)	0.696

*WCS* weekend catch-up sleep, *hs-CRP* high-sensitivity C-reactive protein, *OR* odds ratio, *CI* confidence interval.

^a^Adjusted for age, sex, household income, marital status, economic activity, body mass index, perceived stress, the frequency of binge alcohol consumption, smoking status, medium-intensity physical activity, high-risk for cardiovascular disease, and prevalence of cancer.

Since a significant interaction was found between the WCS group and bedtime inconsistency (p < 0.001), a stratified analysis according to the consistency of bedtime between weekdays and weekends was conducted. A statistically significant reduction in the aORs for the higher quartiles of hs-CRP levels in the moderate WCS group was observed among participants with consistent bedtimes (aOR = 0.88, 95% CI 0.79–0.99 for middle-high hs-CRP levels, aOR = 0.88, 95% CI 0.78–0.99 for the highest hs-CRP levels). Among the participants with inconsistent bedtimes, moderate WCS was not associated with the risk for a higher level of hs-CRP (aOR = 1.12, 95% CI 0.77–1.64 for middle-high hs-CRP levels and aOR = 0.91, 95% CI 0.63–1.33 for the highest hs-CRP levels). Among participants with inconsistent bedtimes, severe WCS showed a significant association with higher hs-CRP levels in comparison to the no-WCS group (aOR = 1.83, 95% CI 1.17–2.85 for middle-high hs-CRP levels and aOR = 1.61, 95% CI 1.04–2.51 for the highest hs-CRP levels). Compared to the no-WCS group, the inverse WCS group showed an increased risk for middle-high hs-CRP levels (aOR = 1.55, 95% CI 1.02–2.35) among the participants with inconsistent bedtimes, while the association was not significant for the highest hs-CRP levels (aOR = 1.18, 95% CI 0.78–1.79) (Table 3).

**Table 3:** Associations between weekend catch-up sleep and high-sensitivity C-reactive protein levels by bedtime consistency.

	Crude OR (95% CI)	*P* value	Adjusted OR^a^ (95% CI)	*p*-value
**Consistent bedtime group (n = 15,951)**				
Middle-low hs-CRP level (vs. lowest)				
No-WCS group	Reference		Reference	
Moderate WCS group	0.78 (0.71,0.86)	< 0.001	0.96 (0.86,1.07)	0.418
Severe WCS group	0.68 (0.57,0.8)	< 0.001	0.96 (0.8,1.16)	0.672
Inverse WCS group	1.20 (0.94,1.53)	0.151	1.27 (0.99,1.63)	0.065
Middle-high hs-CRP level (vs. lowest)				
No-WCS group	Reference		Reference	
Moderate WCS group	0.64 (0.58,0.71)	< 0.001	0.88 (0.79,0.99)	0.026
Severe WCS group	0.52 (0.43,0.62)	< 0.001	0.91 (0.75,1.11)	0.363
Inverse WCS group	0.93 (0.72,1.2)	0.563	1.03 (0.79,1.34)	0.852
Highest hs-CRP level (vs. lowest)				
No-WCS group	Reference		Reference	
Moderate WCS group	0.58 (0.52,0.64)	< 0.001	0.88 (0.78,0.99)	0.027
Severe WCS group	0.49 (0.41,0.59)	< 0.001	0.95 (0.77,1.17)	0.623
Inverse WCS group	0.98 (0.76,1.25)	0.842	1.09 (0.83,1.42)	0.554
**Inconsistent bedtime group (n = 1714)**				
Middle-low hs-CRP level (vs. lowest)				
No-WCS group	Reference		Reference	
Moderate WCS group	1.13 (0.81,1.57)	0.489	1.20 (0.85,1.7)	0.304
Severe WCS group	1.40 (0.93,2.11)	0.108	1.45 (0.94,2.24)	0.089
Inverse WCS group	1.67 (1.15,2.42)	0.008	1.52 (1.03,2.26)	0.037
Middle-high hs-CRP level (vs. lowest)				
No-WCS group	Reference		Reference	
Moderate WCS group	1.12 (0.79,1.59)	0.533	1.12 (0.77,1.64)	0.549
Severe WCS group	1.79 (1.18,2.7)	0.006	1.83 (1.17,2.85)	0.008
Inverse WCS group	1.86 (1.26,2.74)	0.002	1.55 (1.02,2.35)	0.042
Highest hs-CRP level (vs. lowest)				
No-WCS group	Reference		Reference	
Moderate WCS group	0.89 (0.64,1.25)	0.515	0.91 (0.63,1.33)	0.631
Severe WCS group	1.58 (1.06,2.34)	0.024	1.61 (1.04,2.51)	0.034
Inverse WCS group	1.44 (0.99,2.08)	0.058	1.18 (0.78,1.79)	0.436

*WCS* weekend catch-up sleep, *CRP* C-reactive protein, *OR* odds ratio, *CI* confidence interval.

^a^Adjusted for age, sex, household income, marital status, economic activity, body mass index, perceived stress, the frequency of binge alcohol consumption, smoking status, medium-intensity physical activity, high-risk for cardiovascular disease, and prevalence of cancer.

**Figure 2:**
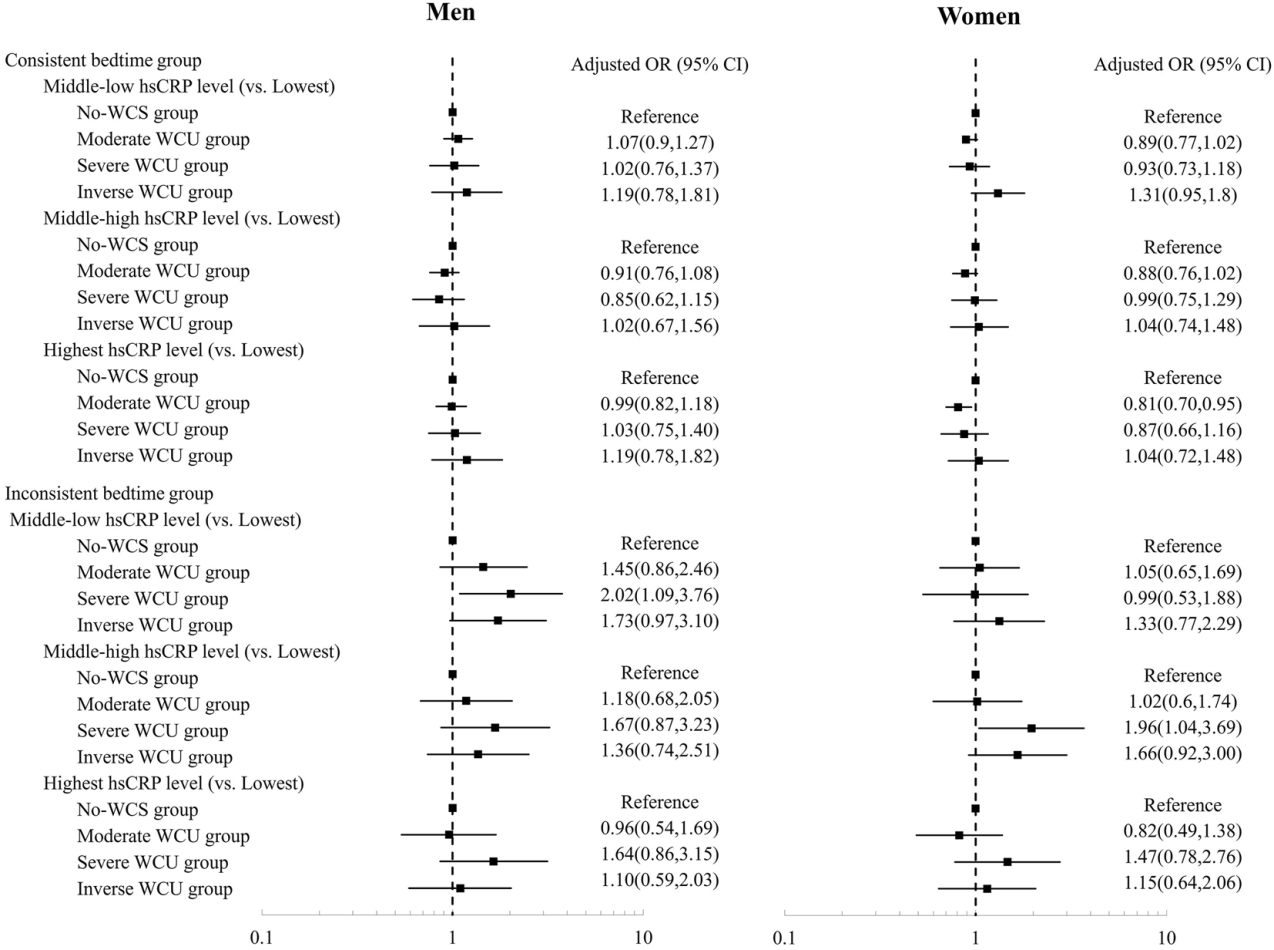
Stratified analysis of the association between weekend catch-up sleep and serum high-sensitivity C-reactive protein levels by bedtime consistency according to sex. *OR* odds ratio, *CI* confidence interval, *hsCRP* high-sensitivity C-reactive protein.

Both sexes generally showed similar results in the analysis stratified according to bedtime consistency; however, only women showed a protective effect of moderate WCS among the participants with consistent bedtimes (aOR = 0.88, 95% CI 0.76–1.02 for middle-high hs-CRP levels and aOR = 0.81, 95% CI 0.70–0.95 for the highest hs-CRP levels) and a higher risk associated with severe WCS among the participants with inconsistent bedtimes (aOR = 1.96, 95% CI 1.04–3.69 for middle-high hs-CRP levels and aOR = 1.47, 95% CI 0.78–2.76 for the highest hs-CRP levels) (Fig. 2).

**Figure 3:**
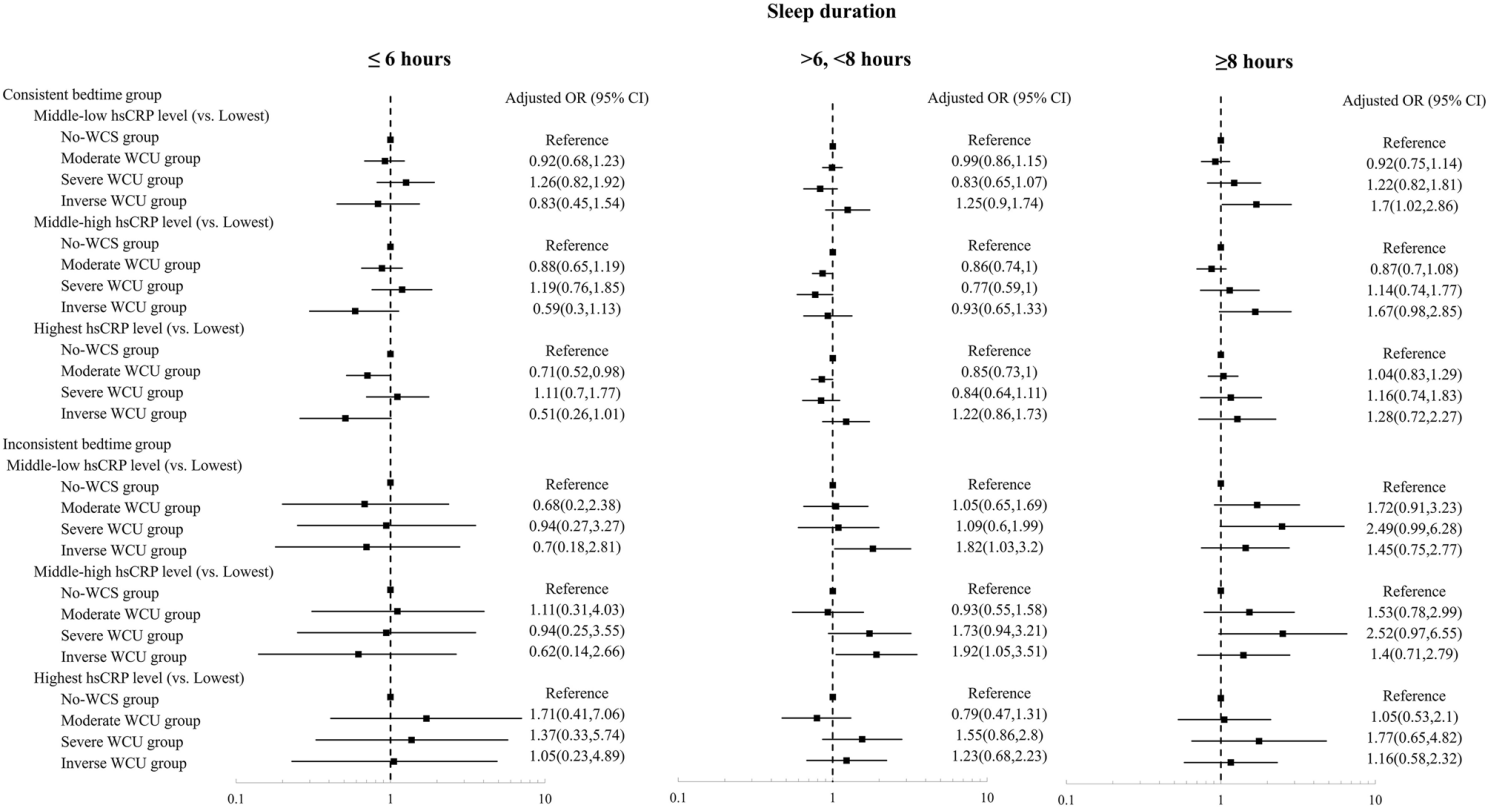
Stratified analysis of the association between weekend catch-up sleep and serum high-sensitivity C-reactive protein levels by bedtime consistency according to sleep duration. *OR* odds ratio, *CI* confidence interval, *CRP* C-reactive protein.

When stratified by sleep duration, a significantly lower aOR for the highest hs-CRP level (versus the lowest level) was found in participants with WCS who had sleep durations of ≤ 6 h (aOR = 0.71, 95% CI 0.52–0.98), and in participants with WCS who had sleep durations of 6–8 h (aOR = 0.85, 95% CI 0.73–1.00) (Fig. 3).

**Figure 4:**
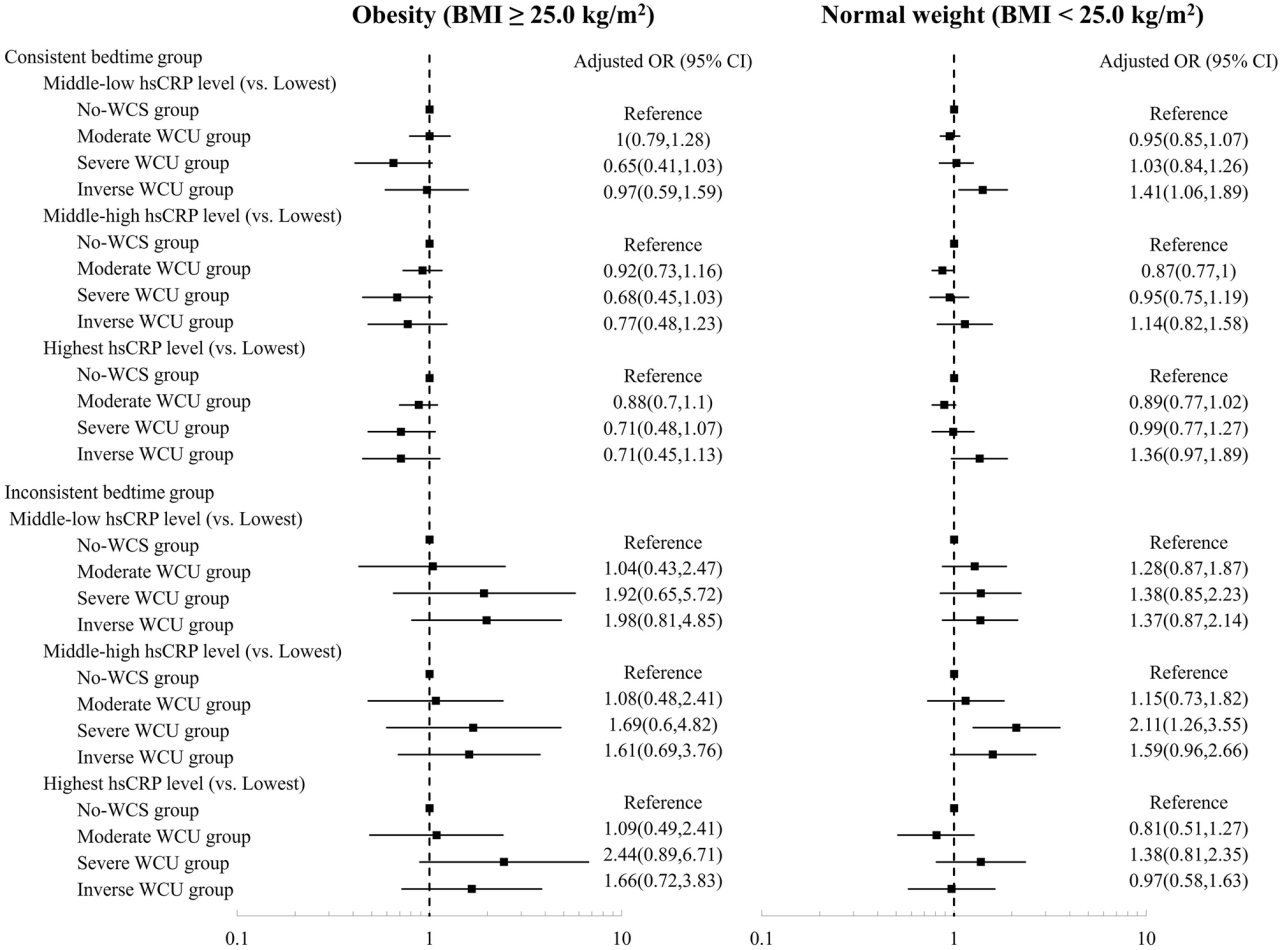
Stratified analysis of the association between weekend catch-up sleep and serum high-sensitivity C-reactive protein levels by bedtime consistency according to obesity. *OR* odds ratio, *CI* confidence interval, *hsCRP* high-sensitivity C-reactive protein.

Figure 4 shows the results of the stratified analysis for the association between WCS and hs-CRP levels by bedtime consistency according to obesity. A protective effect of moderate WCS was observed in participants with a consistent bedtime and normal weight (aOR = 0.87, 95% CI 0.77–1.00 for middle-high hs-CRP levels and aOR = 0.89, 95% CI 0.77–1.02 for the highest hs-CRP levels). For the inconsistent bedtime group, an increased risk was shown in point estimates for both the obesity and normal weight groups; however, statistical significance was generally not observed.

**Figure 5:**
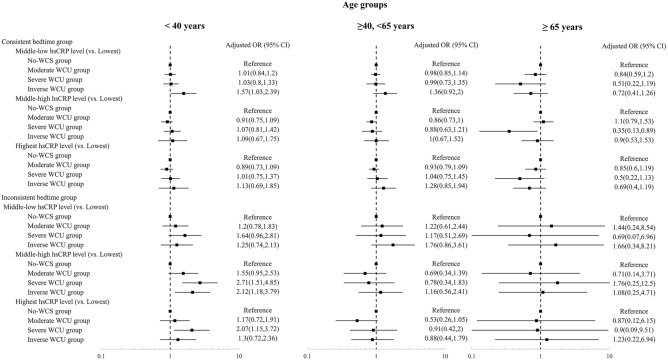
Stratified analysis of the association between weekend catch-up sleep and serum high-sensitivity C-reactive protein levels by bedtime consistency according to age group. *OR* odds ratio, *CI* confidence interval, *hsCRP* high-sensitivity C-reactive protein.

According to the subgroup analysis based on age group, a protective effect of moderate WCS was observed in participants aged 40–64 with consistent bedtimes (aOR = 0.86, 95% CI 0.73–1.00 for middle-high hs-CRP levels and aOR = 0.93, 95% CI 0.79–1.09 for the highest hs-CRP levels). An association of increased risk and severe WCS was found in the participants under 40 years (aOR = 2.71, 95% CI 1.51–4.85 for middle-high hs-CRP levels and aOR = 2.07, 95% CI 1.15–3.72 for the highest hs-CRP levels). Among older participants with consistent bedtimes, a protective effect of severe WCS for middle-high hs-CRP levels was shown (aOR = 0.35, 95% CI 0.13–0.89). (Fig. 5).

Although statistical significance was not observed in the linear regression analyses, the beta estimation of log-CRP decreased in the moderate WCS group, similar to the results obtained using multinomial logistic regression. In women, log-CRP decreased in the moderate, severe, and inverse WCS groups compared to the no-WCS group, and log CRP decreased the most in the moderate WCS group. Statistical significance was also confirmed in women. In an analysis according to bedtime inconsistency, log-CRP tended to decrease in participants with consistent bedtimes and increased in the severe and inverse WCS groups with inconsistent bedtimes (Supplement Table S1).

## Discussion

This analysis of nationwide population-based cross-sectional data was conducted to investigate the association of WCS with hs-CRP levels and its interaction with bedtime inconsistency. Our results show that moderate WCS was associated with lower hs-CRP levels, with the main contribution to this relationship stemming from the participants with consistent bedtimes between weekdays and weekends. In participants with inconsistent bedtimes, severe WCS and inverse WCS were associated with increased risks of having hs-CRP levels in the higher quartiles in comparison with the no-WCS group. When stratified according to sex, the association of WCS with higher levels of hs-CRP was more pronounced in women.

Our findings on the relationship between recovery sleep and hs-CRP as an inflammation marker correspond to the results of previous studies^15,17,19^. A clinical trial in American adults reported that mild sleep restriction raised 24-h IL-6 levels, while recovery sleep reduced cortisol levels compared with baseline^19^. Since IL-6 is also a representative marker of inflammation, and cortisol is known to be an anti-inflammatory hormone, these results are consistent with those of our study. Two recently published studies using KNHANES data have reported that a short duration of WCS was associated with lower hs-CRP levels. Jung et al. showed that at least 1 h and less than 2 h of WCS was associated with a reduced risk of elevated hs-CRP levels (1 mg/L or more) in Korean adult workers^17^. Han et al. found that WCS of less than 3 h was associated with a reduced risk of elevated hs-CRP levels in Korean adults aged 19 years or older, and the results were consistent in participants who slept for less than 6 h and in non-obese participants^15^.

A possible mechanism underlying the relationship between recovery sleep and hs-CRP levels has been suggested. Sleep deprivation or sleep disturbances that raise catecholamine levels could cause dysregulated autonomic function, affecting inflammation-related gene expression due to adrenergic signaling and the subsequent production of inflammation markers^2,25^. Sleep deprivation could also change the levels of several hormones; for example, it may lead to increased levels of cortisol and reduced levels of melatonin, which could lead to chronic metabolic diseases such as hyperinsulinemia, obesity, and high blood pressure^19,26^. Moderate WCS stabilizes the autonomic nervous system and hormonal imbalance, resulting in beneficial health effects that may manifest as reductions in the parameter of hs-CRP.

When stratified according to bedtime inconsistency, we found that moderate WCS was not associated with lower hs-CRP levels, while in contrast, severe WCS was significantly associated with higher hs-CRP levels only among participants with bedtime inconsistency. That is, the previously suggested protective effect of moderate WCS was not shown, and increased risks were observed in severe WCS. Intraindividual variability in sleep duration or inconsistent bedtimes disrupts homeostasis, which may increase inflammation. Although no previous studies have yet assessed the effect of WCS and bedtime inconsistency on inflammation, a U.S. study showed that several sleep inconsistency-related variables (time in bed, number of nightly awakenings, terminal wakefulness, etc.) were related to inflammatory dysfunction, measured in terms of CRP and IL-6 levels^27^. Similarly, in the present study, participants who did not maintain sleep homeostasis between weekdays and weekends in terms of duration and bedtime showed higher hs-CRP levels. A similar indicator of bedtime inconsistency is social jetlag, which measures the sleep midpoint discrepancy between workdays and free days. Previous studies have shown that social jetlag is associated with metabolic syndrome biomarkers (e.g., waist circumference and fat mass), cardiovascular risk profile (e.g., blood pressure and resting heart rate) and endocrine profile (e.g., fasting cortisol levels)^28,29^. Based on the Donedin study, interestingly, in a subgroup analysis, social jetlag in the metabolically unhealthy obese group showed a statistically significant relationship with hs-CRP, a marker of inflammation^28^. Huang et al. examined the association between cardiovascular events, which are known to be inflammation-related conditions, and metabolic abnormalities and sleep regularity using 7 days of the SD of sleep duration or sleep-onset time in prospective settings, and found separate associations for an increased risk of cardiovascular events and metabolic abnormalities^30,31^.

In the inverse WCS group, participants with inconsistent bedtimes showed significantly higher risks for middle-low and middle-high hs-CRP levels, but not for the highest hs-CRP levels. Our findings might suggest that the inverse WCS group should be separately considered when assessing the risk for inflammation. To generate high-level evidence, further studies are warranted to confirm the association between sleeping less on the weekend and a higher risk of inflammation.

In this study, significant results were observed for women, whereas the associations were inconclusive in men. Although the factors that could explain this difference are not well-known, sex differences in the relationship between sleep-related factors and inflammation have been reported in previous studies. According to an earlier study conducted in Korea, difficulty in initiating sleep, difficulty in maintaining sleep, and poor quality of sleep were significantly associated with higher hs-CRP levels in women^32^. In a study conducted in China, He et al. reported that long sleep duration (≥ 9 h) was associated with elevated hs-CRP levels only in women^33^. The previously mentioned study conducted in the U.S. also showed a significant relationship between latent sleep inconsistency factors and inflammation for women, but not men^27^. In contrast, a population-based study in the U.S. reported an association between short sleep duration (≤ 6 h) and significantly higher CRP levels only in men^34^. Our findings that women with moderate WCS and consistent bedtimes were more likely to have lower levels of hs-CRP, and that women with severe WCS and inconsistent bedtimes had higher levels of hs-CRP, might reflect biological differences, including the role of sex hormones. Androgens, which are present at higher levels in post-pubertal men than in women, suppress pro-inflammatory responses. For example, studies have shown that men with subnormal testosterone had higher levels of inflammatory markers (such as tumor necrosis factor-α). Estrogen enhanced the production of pro-inflammatory cytokines at low concentrations, as in post-menopausal women, and reduced their production at high doses, as in pregnant women or at varying points during the menstrual cycle^35^. This difference in the pro-inflammatory response to estrogen according to female age may partially explain the statistical significance found for women in this study. Furthermore, gender-related differences in social roles (e.g., working), and health habits (e.g., smoking and drinking alcohol) might contribute to these differences.

We conducted a stratified analysis of sleep duration and obesity; the protective effect of moderate-WCS was consistent in the group with normal weight or 6–8 h of sleep duration with a consistent bedtime. As sleep duration is a critical factor, many previous studies have reported an increase in inflammatory markers with sleep deprivation and/or excess^3,32,36^. BMI is also a strong risk factor for cardiovascular disease, and the relationship between increased BMI and inflammation is well known^37,38^. The protective effect of moderate WCS was not observed in those with other risk factors, including long sleep duration or obesity. A partial interpretation of this finding is that obesity and sleep duration might have had a stronger influence. Similar to our results, Han et al., showed that a lower risk for high hs-CRP levels among the WCS group was not found in obese people and those with longer weekday sleep duration^15^.

A subgroup analysis according to age group was conducted because age itself is associated with hs-CRP levels. Jung et al., reported that elevated risks of high hs-CRP were observed in participants aged 40–64 years and ≥ 65 years in comparison with those aged < 40 years, with an increasing tendency according to age^17^. A protective effect of moderate WCS was found in participants aged 40–64 with consistent bedtimes, as in the main analysis. Our results are in line with those of a previous study showing that WCS was associated with a reduced risk of metabolic syndrome among middle-aged (35–60 years) Koreans with chronic sleep insufficiency^39^, and hs-CRP was suggested as a possible mechanism to explain the above relationship. Severe WCS seems to be associated with a protective effect in older adults with consistent bedtimes. However, this association was not observed in other age groups, which is similar to the results reported by Han et al. that longer WCS (more than 3 h) was not associated with beneficial effects on hs-CRP levels. The reasons for the association of severe WCS with lower hs-CRP levels in the older group are not clear. However, the difference in employment status by WCS groups between age groups might affect this association. Unlike other age groups, among older adults, the proportion of the employed in the severe WCS group (59.0%) was about twice that in the no-WCS group (31.2%). The healthy worker effect could have remained even though we adjusted for employment status in multiple models.

Our study has several strengths. We used a nationally representative sample from South Korea (KNHANES VII), which permits generalization of the results to the overall population of South Korea. With a sufficient sample size, detailed information for sleep duration and bedtime, and exact measurements of hs-CRP, it was possible to obtain meaningful results regarding the effects of WCS and sleeping less on the weekend on hs-CRP, interactions of those associations with bedtime inconsistency, and sex differences in the observed relationships.

However, precautions should be taken regarding the interpretation of the results due to certain limitations. The cross-sectional nature of the study design means that causal or temporal relationships could not be determined. In particular, severe WCS could be the result of physical and mental illnesses (e.g., hypertension, diabetes, infectious diseases, or depression) that cause fatigue and have well-known relationships with inflammation. It is possible that individuals with those conditions may require longer recovery sleep on weekends. In particular, acute infections, chronic infectious diseases, autoimmune diseases, Alzheimer's disease, and Parkinson's disease could have influenced the hs-CRP levels^40^, and we could not adjust for these conditions in our study, because information on comorbidities or history of these diseases had not been collected in the database. Data on sleep duration and bedtime were obtained by self-reported questionnaires, not by an objective method. According to Lockley et al., there is a close correlation between subjective and actigraphic measurements of sleep and sleep rhythms, especially for sleep timing and duration^41^. Sleep diaries and direct measurements are better methods for obtaining data on sleep patterns, and recall bias due to our use of questionnaires cannot be excluded. Given that the information on sleep quality, sleep pattern changes, sleep-related diseases including insomnia, and utilization of medications related to sleep (e.g., hypnotics and sedative antidepressants) were not recorded in the KHANES, we could not consider these possible confounders in the analysis. Furthermore, it was not possible to consider factors such as a recent history of surgery or infection, which could have temporarily increased hs-CRP levels.

## Conclusion

In conclusion, we found that the association of WCS with hs-CRP levels differed according to bedtime inconsistency between weekdays and weekends. The lowered hs-CRP level observed in the moderate WCS group was mainly due to participants with consistent bedtimes, whereas this relationship was not observed in participants with inconsistent bedtimes. Furthermore, a trend for higher hs-CRP levels was found in the severe and inverse WCS groups among participants with inconsistent bedtimes. Our findings provide evidence that the effect of WCS on hs-CRP levels should be interpreted in light of bedtime inconsistency in an Asian population.

## Supplementary Information

Supplementary Table S1.

## Data Availability

The data used in this study are openly available from the Korea National Health and Nutritional Examination Survey webpage (URL: http://knhanes.cdc.go.kr/knhanes/index.do).
