# Supplementary material for: Effect of weekend catch-up sleep on high-sensitivity C-reactive protein levels according to bedtime inconsistency: a population-based cross-sectional study
Source: Sci Rep. 2022 Dec 14;12:21619. doi: 10.1038/s41598-022-25787-x (PMC9751111; doi:10.1038/s41598-022-25787-x)
Supplement: Supplementary file 1 — Supplementary Table S1. [file 41598_2022_25787_MOESM1_ESM.docx]

**Supplement table S1. Linear regression analysis of the associations between weekend catch-up sleep and logarithm of high-sensitivity C-reactive protein**

|  | **Simple regression model** | | | **Multiple regression model^a^** | | |
| --- | --- | --- | --- | --- | --- | --- |
|  | **beta coefficient** | **Standard error** | ***p*-value** | **beta coefficient** | **Standard error** | ***p*-value** |
| **Total (n=17,665)** |  |  |  |  |  |  |
| No-WCS group | Reference |  | <.001 | Reference |  | 0.138 |
| Moderate WCS group | -0.169 | 0.016 |  | -0.035 | 0.016 |  |
| Severe WCS group | -0.179 | 0.027 |  | 0.005 | 0.026 |  |
| Inverse WCS group | -0.050 | 0.031 |  | -0.019 | 0.029 |  |
| **Men (n=7,948)** |  |  |  |  |  |  |
| No-WCS group | Reference |  | 0.002 | Reference |  | 0.817 |
| Moderate WCS group | -0.137 | 0.024 |  | -0.010 | 0.024 |  |
| Severe WCS group | -0.144 | 0.040 |  | 0.029 | 0.040 |  |
| Inverse WCS group | -0.046 | 0.047 |  | 0.001 | 0.045 |  |
| **Women (n=9,717)** |  |  |  |  |  |  |
| No-WCS group | Reference |  | <.001 | Reference |  | <.001 |
| Moderate WCS group | -0.188 | 0.021 |  | -0.052 | 0.021 |  |
| Severe WCS group | -0.209 | 0.036 |  | -0.018 | 0.036 |  |
| Inverse WCS group | -0.052 | 0.042 |  | -0.026 | 0.039 |  |
| **Consistent bedtime group (n=15,951)** |  |  |  |  |  |  |
| No-WCS group | Reference |  | <.001 | Reference |  | 0.415 |
| Moderate WCS group | -0.172 | 0.017 |  | -0.028 | 0.017 |  |
| Severe WCS group | -0.223 | 0.030 |  | -0.007 | 0.030 |  |
| Inverse WCS group | -0.053 | 0.040 |  | -0.016 | 0.038 |  |
| **Inconsistent bedtime group (n=1,714)** |  |  |  |  |  |  |
| No-WCS group | Reference |  | 0.024 | Reference |  | 0.232 |
| Moderate WCS group | -0.039 | 0.058 |  | -0.035 | 0.054 |  |
| Severe WCS group | 0.111 | 0.067 |  | 0.084 | 0.063 |  |
| Inverse WCS group | 0.113 | 0.062 |  | 0.036 | 0.059 |  |

WCS: weekend catch-up sleep; hs-CRP: high-sensitivity C-reactive protein;

^a^Adjusted for age, sex, household income, marital status, economic activity, body mass index, perceived stress, the frequency of binge alcohol consumption, smoking status, medium-intensity physical activity, high-risk for cardiovascular disease, and prevalence of cancer.
